# Draft Genome Sequence of *Arthrobacter* sp. Strain SPG23, a Hydrocarbon-Degrading and Plant Growth-Promoting Soil Bacterium

**DOI:** 10.1128/genomeA.01517-15

**Published:** 2015-12-23

**Authors:** Panagiotis Gkorezis, Eric M. Bottos, Jonathan D. Van Hamme, Sofie Thijs, Francois Rineau, Andrea Franzetti, Maria Balseiro-Romero, Nele Weyens, Jaco Vangronsveld

**Affiliations:** a Centre for Environmental Sciences, Hasselt University, Diepenbeek, Belgium; b Department of Biological Sciences, Thompson Rivers University, Kamloops, British Columbia, Canada; c Department of Environmental Sciences, University of Milano-Bicocca, Milan, Italy; d Department of Soil Science and Agricultural Chemistry, University of Santiago de Compostela, Campus Vida, Santiago de Compostela, Spain

## Abstract

We report here the 4.7-Mb draft genome of *Arthrobacter* sp. SPG23, a hydrocarbonoclastic Gram-positive bacterium belonging to the *Actinobacteria*, isolated from diesel-contaminated soil at the Ford Motor Company site in Genk, Belgium. Strain SPG23 is a potent plant growth promoter useful for diesel fuel remediation applications based on plant-bacterium associations.

## GENOME ANNOUNCEMENT

Members of the genus *Arthrobacter* have been associated with the degradation of compounds, such as 4-chlorophenol (1), polychlorinated biphenyls (2, 3), pentachloronitrobenzene, (4), 2-nitrobenzoate (5), atrazine (6), 2,4-dinitrotoluene (7), *para*-nitrophenol (8), 4-bromophenol (9), phenanthrene, and phthalates (10, 11). Genome sequence data indicate that several strains of *Arthrobacter* are capable of degrading aromatic compounds, including *Arthrobacter* sp. YC-RL1 (12), *Arthrobacter* sp. W1 (13), and *Arthrobacter* sp. strain SJCon (14).

Using gas chromatography (GC) (model 450; Agilent Technologies) coupled to mass spectrometry (MS) (model 220; Agilent Technologies), *Arthrobacter* sp. SPG23 was found to degrade up to 25% of diesel range organics over 10 days. Partial 16S rRNA gene sequence data and phenotypic profiling indicate that SPG23 is related to *Arthrobacter* sp. FB24 (GenBank accession no. CP000454).

For sequencing, genomic DNA was extracted with a Qiagen blood and tissue kit (Qiagen NV, Hilden, Germany), and an Ion Torrent PGM was used to generate a whole-genome shotgun using methods described by Thijs et al. (15).

In total, 912,912 reads (mean length, 214 bases) generated 196 Mb of data in Torrent suite 4.2.1, which were assembled into 38 contigs using MIRA 4.0.5 (16), giving a consensus length of 4,703,830 bp at 40.0× coverage (largest contig, 432,818 bp; *N*_50_, 216.99 bp). Open reading frame (ORF) prediction and gene annotation were carried out using the PGAP (NCBI) pipeline (17). The contigs were ordered in Mauve (18) using the *Arthrobacter* sp. FB24 genome as a reference.

The SPG23 genome consists of a single circular chromosome (66.3% G+C content), including 3,902 coding genes that were arranged into pathways using Pathway Tools (19, 20), 280 pseudogenes, 15 rRNAs (5S, 16S, and 23S), 52 tRNAs, and 1 noncoding RNA (ncRNA).

Annotation has predicted gene-coding clusters for alkane degradation (5 genes), benzene degradation (9 genes), and naphthalene (8 genes). Genes for plant growth-promoting traits are present, affirming the results from phenotypic assays that determined that these are involved in symbiotic nitrogen fixation, siderophore biosynthesis, and inorganic phosphorous solubilization and uptake.

Based on both its hydrocarbon-degrading and plant growth-promoting capacities, *Arthrobacter* sp. SPG23 is a promising candidate as an inoculant to stimulate the phytoremediation of petroleum-contaminated sites.

### Nucleotide sequence accession numbers.

This whole-genome shotgun project has been deposited at DDBJ/EMBL/GenBank under the accession number JYCN00000000. The version described in this paper is version JYCN01000000.
